# Influence of Cerium Oxide Nanoparticles on Two Terrestrial Wild Plant Species

**DOI:** 10.3390/plants10020335

**Published:** 2021-02-10

**Authors:** Daniel Lizzi, Alessandro Mattiello, Alessio Adamiano, Guido Fellet, Emanuele Gava, Luca Marchiol

**Affiliations:** 1 DI4A—Department of Agriculture, Food, Environment and Animal Sciences, University of Udine, Via delle Scienze 206, 33100 Udine, Italy; lizzi.daniel1@spes.uniud.it (D.L.); alessandro.mattiello@uniud.it (A.M.); guido.fellet@uniud.it (G.F.); 2 Department of Life Sciences, University of Trieste, Via Licio Giorgieri 10, 34127 Trieste, Italy; 3 Institute of Science and Technology for Ceramics (ISTEC), National Research Council (CNR), Via Granarolo 64, 48018 Faenza, Italy; alessio.adamiano@istec.cnr.it; 4 Laboratory of Inorganic Micro Pollutants, Regional Environmental Protection Agency of Friuli Venezia Giulia (ARPA-FVG), Via Colugna 42, 33100 Udine, Italy; emanuele.gava@arpa.fvg.it

**Keywords:** cerium oxide nanoparticles, *Holcus lanatus*, *Diplotaxis tenuifolia*, biometric variables, Ce accumulation

## Abstract

Most current studies on the relationships between plans and engineered nanomaterials (ENMs) are focused on food crops, while the effects on spontaneous plants have been neglected so far. However, from an ecological perspective, the ENMs impacts on the wild plants could have dire consequences on food webs and ecosystem services. Therefore, they should not be considered less critical. A pot trial was carried out in greenhouse conditions to evaluate the growth of *Holcus lanatus* L. (monocot) and *Diplotaxis tenuifolia* L. DC. (dicot) exposed to cerium oxide nanoparticles (*n*CeO_2_). Plants were grown for their entire cycle in a substrate amended with 200 mg kg^−1^ *n*CeO_2_ having the size of 25 nm and 50 nm, respectively. *n*CeO_2_ were taken up by plant roots and then translocated towards leaf tissues of both species. However, the mean size of *n*CeO_2_ found in the roots of the species was different. In *D. tenuifolia*, there was evidence of more significant particle aggregation compared to *H. lanatus*. Further, biomass variables (dry weight of plant fractions and leaf area) showed that plant species responded differently to the treatments. In the experimental conditions, there were recorded stimulating effects on plant growth. However, nutritional imbalances for macro and micronutrients were observed, as well.

## 1. Introduction

Nanotechnology has revolutionized the manufacturing aspects in several fields of applications. The tunable chemical and physical properties of engineered nanomaterials (ENMs) are still improving electronics, energy, aerospace, human health, innovative materials, and many others [1]. Despite the current and expected great benefits of nanotechnologies, an unintended side effect is the uncontrolled release of ENMs. Due to involuntarily releases, tons of ENMs are discharged into the environment [2]. According to quantitative models, soils and freshwaters are the endpoints of such materials [3,4,5]. Such evidence raises concern on the impact of ENMs on the ecosystems and biota since we still have patchy knowledge on this issue [6].

Cerium oxide nanoparticles (*n*CeO_2_) are included in the top 10 most produced and widely utilized nanomaterials globally; their global estimated production is 10,000 metric tons per year [7]. The global emission of *n*CeO_2_ in soil was estimated to be 129–1029 metric tons per year [8]. The impact of *n*CeO_2_ is already under observation in different environmental matrices and ecosystems [9,10]. It has been reported a *n*CeO_2_ concentration in soil in the range 0.09–1.12 mg kg^−1^ [11].

So far, data are available in the scientific literature regarding the adverse effects on plant metabolism and growth due to ENMs exposure [12,13,14]. On the other hand, convincing demonstrations of the positive effects of ENMs applied to plants as fertilizers were already provided [15]. However, due to the vastness of the investigation field, the knowledge acquired is still instead fragmentary for some reasons. Early studies were carried out without specific reference protocols and different conditions (e.g., hydroponics, synthetic soil, potting soil, or agricultural soil), obtaining different results. For obvious reasons (e.g., food and fodder production, agriculture management, and social and financial issues), most studies have been conducted on food crops [16].

Concerning *n*CeO_2_—plants relationships, investigations have been carried out in short-term experiments and controlled conditions (i.e., Petri dishes and hydroponic systems) on germinating seeds or very young plant specimens [17]. In these experimental conditions, studies have been carried out on *Triticum aestivum* [18], *Zea mays* [19], *Hordeum vulgare* [20,21], *Glycine max* [22], *Cucumber sativus* [23], *Raphanus sativus* [24], and *Lycopersicum esculentum* [25,26,27,28,29].

From a wider perspective a tiny number of plant species was investigated so far. In fact, crops represent about 0.1% of the vascular plants known to science [30]. Spontaneous plant species are not less important. Being soil one of the endpoints of ENMs, it is of the utmost importance to consider the interactions with plants, to be intended not only as food producers but as primary producers and ecosystem services providers [31].

Beyond crops, ENMs-plant relationships were investigated on some spontaneous species. More aquatic and wetland species have been studied than terrestrial ones [32,33,34]. Regarding terrestrial ecosystems, as far as we know, *Pinus sylvestris* L. and *Quercus robur* L. are the only no-food terrestrial plant species investigated for the exposure to respectively nano-Ag and *n*CeO_2_ [35].

The aim of this work is to evaluate the influence of *n*CeO_2_ with different particle size on the entire growth cycle of *Holcus lanatus* (L.) and *Diplotaxis tenuifolia* (L.) (DC) (dicot). The grass *H. lanatus* (monocot) is a hairy, tufted, fibrous-rooted and meadow soft perennial grass, growing between 50 and 100 cm tall, belonging to Poaceae family [36]. It occurs over a wide range of soil types and fertility conditions. It is characterized by the absence of rhizomes and it spreads by developing new shoots and roots at its nodes and forming a blanket of runners on the soil surface. The dicot *D. tenuifolia* is a Brassica perennial flowering plant, native in Europe and West Asia. It is commonly found in different habitats but in particular in ruderal plant associations, in abandoned fields and ruderal areas, more rarely in cultivated fields [37]. The species have been chosen since they are widespread in natural systems, highly competitive, and easily adaptable to different ecological conditions.

## 2. Results

### 2.1. Nanomaterial Characterization

**Figure 1:**
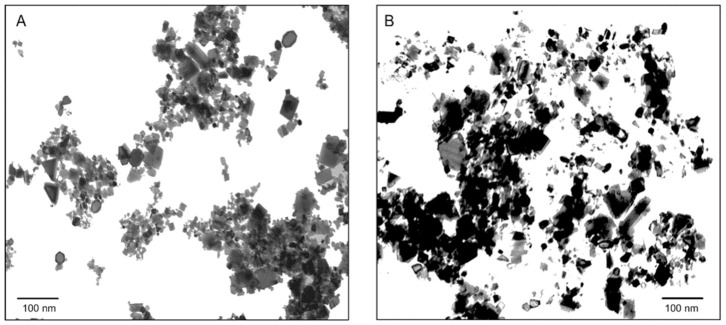
TEM image of *n*CeO_2_ 25 nm (**A**) and 50 nm (**B**) suspensions.

The hydrodynamic diameter (Hd) distribution of both *n*CeO_2_ 25 nm and 50 nm is in agreement with the value provided by the supplier. Both materials exhibit a monodisperse size particle distribution in the nanometric range with relatively low polydispersity index (PDI) (0.17 and 0.25) and the main size peak at 62.0 nm and 91.0 nm, respectively. The relative Z-averages sizes were found to be much larger than these values (127 nm and 206 nm, respectively, *n*CeO_2_ 25 nm and 50 nm), indicating particle aggregation. Since a high net surface charge is typically associated with weak nanoparticles interactions and aggregation, these data are coherent with the higher Z-average size detected for sample *n*CeO_2_ 50 nm with respect to *n*CeO_2_ 25 nm (ζ–potentials 39.2 mV (25 nm) and 24.1 mV (50 nm)). Particle field observations of *n*CeO_2_ suspensions are reported in Figure 1.

### 2.2. nCeO_2_ in Plant Tissues

**Figure 2:**
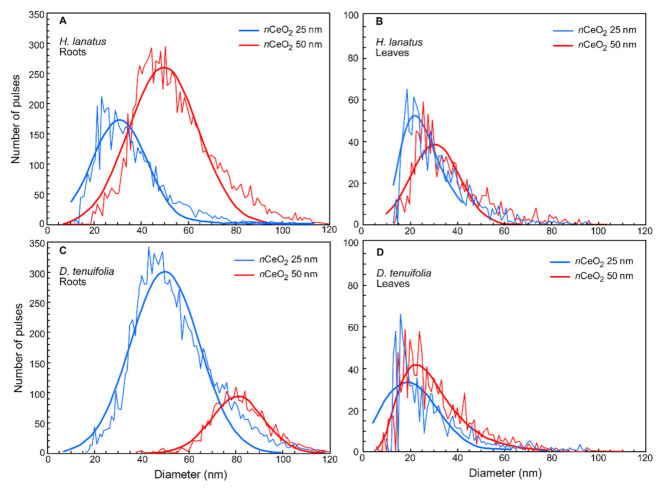
Particle size distribution of *n*CeO_2_ 25 nm (blue) and 50 nm (red) extracted by enzymatic digestion procedure from roots (**A**) and leaves (**B**) of *H. lanatus*, and from roots (**C**) and leaves (**D**) of *D. tenuifolia*.

Before illustrating and discussing the experimental results, it is necessary to prove whether the *n*CeO_2_ has been taken up by plant roots and possibly translocated towards the aerial parts. Plants grew in the presence of different concentrations of *n*CeO_2_ in the soil medium. The early step of this study was to verify the entry of *n*CeO_2_ into plant tissues and verify if any particle size transformations into the plant tissues occurred. Roots and leaves of *H. lanatus* and *D. tenuifolia* were subjected to the enzyme extraction procedure, and sp-ICP-MS analyzed the extracts. The data obtained from the sp-ICP-MS analysis, reported in Figure 2 and Table S1, provide two early results.

The first is that *n*CeO_2_ were found in the root tissues and leaves of both species. The second is that *n*CeO_2_ root uptake occur differently in the two species, with consequences on *n*CeO_2_ size and the dissolution of Ce ions inside the leaf tissues. In particular, in the roots of *H. lanatus* the most frequent *n*CeO_2_ size (30 nm and 51 nm) and the mean particle size (36 nm and 56 nm) were very close to the actual size of Ce nanoparticles (25 nm and 50 nm, respectively) (Figure 2A and Table S1). After entering the root tissues, *n*CeO_2_ has not undergone aggregation and could move to the aerial plant fractions through the vascular tissues. A small fraction of dissolved Ce ion (7.07 µg L^−1^) was detected in the root extracts containing the larger *n*CeO_2_, whereas it was practically absent in plants treated with *n*CeO_2_ 25 nm (Table S1). In roots of *D. tenuifolia* the right-shift of fitting curves (Figure 2C) compared to previous ones indicate particle aggregation. In fact, the most frequent particle size was 50 nm and 79 nm while the mean particle size was 53 nm and 82 nm that are values higher than the reference size, 25 nm and 50nm (Figure 2C and Table S1). By observing the curves reported in Figure 2A–C, we can appreciate that in the roots of *D. tenuifolia* was detected a much greater number of small *n*CeO_2_ and a much smaller number of larger particles compared to *H. lanatus*.

*n*CeO_2_ were also extracted from leaf tissues of both species (Figure 2B–D). However, the sp-ICP-MS analyses showed that fewer particles were accumulated in the leaves than the ones found in the roots, but they were also smaller in size (range 26–36 nm) (Figure 2B–D and Table S1). Finally, a negligible concentration of dissolved Ce was found in the leaf extracts for both species. So, such smaller *n*CeO_2_ did not undergo dissolution after having reached the aerial plant fractions (Table S1).

### 2.3. Plant Growth

The following biometric variables were detected on the fractions of *H. lanatus* and *D. tenuifolia*: root dry matter, number of stems per plant, stems dry matter, leaves dry matter, leaf area per plant, and total plant dry matter. A two-way ANOVA was carried out to evaluate the effects of the experimental factors on the mentioned plant growth variables (Table S2).

An overall view of the results shows clearly that the response of the species to *n*CeO_2_ was different. For the biometric variables, except leaves dry mass, a statistically significant effect of the factor “species” was observed. A statistically significant interaction “species X treatment” on root dry matter (*p* = 0.0368 *) and the number of stems per plant (*p* = 0.0157 *) were observed, as well.

The aerial biomass production of plants responded to the presence of *n*CeO_2_. The effect of the “treatment” factor was statistically significant in the case of the number of stems per plant (*p* = 0.0094 **), for the leaf area (*p* = 0.0005 **) and the dry weight of the leaves (*p* = 0.0482 *). The effect of the treatment on the dry weight of the stems was not statistically significant. However, a *p*-value of 0.0574—very close to the significance threshold—indicates that this variable is consistent with the previous evidence (Table S2).

**Figure 3:**
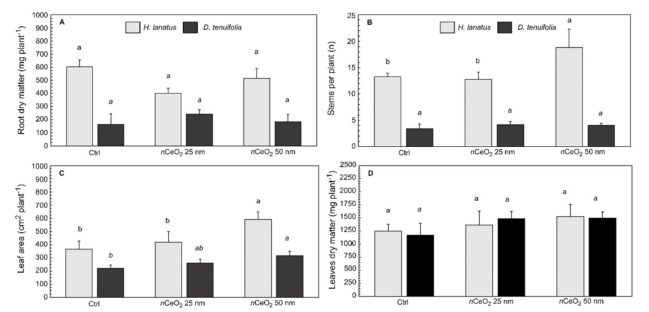
(**A**) Root dry matter, (**B**) number of stems per plant, (**C**) leaf area and (**D**) leaf dry matter ± standard deviation of *H. lanatus* and *D. tenuifolia*. Comparison between control and plants grown in presence of 200 mg kg^−1^ *n*CeO_2_ having respectively 25 nm and 50 nm. For each species the statistically significant difference (*p* ≤ 0.05) between treatments is indicated by the letters using one-way ANOVA follow by Tukey’s test.

The root dry mass, number of stems per plant, leaf area, and leaves dry mass per plant are presented in Figure 3A–D. The results of the post-hoc comparisons Tukey HSD test for each species are reported, as well.

There was no significant change in the root dry mass of *H. lanatus* (*p* = 0.1154) and D. tenuifolia (*p* = 0.3096) in response to the treatments (Figure 3A). This is likely due to the buffer effect of the potting soil, which—at the not very high concentration of 200 mg kg^−1^ *n*CeO_2_—hid any effect on the root growth. Figure 3B reports the number of stems per plant. Tillering, i.e., the production of secondary stems, is a very important phenological phase of monocotyledon species. In addition to producing a large number of secondary stems compared to *D. tenuifolia*, *H. lanatus* was also clearly stimulated by *n*CeO_2_ 50 nm, which supported the increase of stem number per plant by 31% (*p* = 0.0269 *).

Since the treated plants had a greater vegetative development than the control ones, it is reasonable to expect an increase in leaf apparatus. This has happened in terms of leaf area (Figure 3C). In *H. lanatus*, the treatment effect with *n*CeO_2_ 25 nm was not intense enough to be statistically detectable by the ANOVA. In contrast, *n*CeO_2_ 50 nm resulted in an increase of 66% and 41% for the control and *n*CeO_2_ 25 nm treated plants, respectively. In *D. tenuifolia* the response to *n*CeO_2_ treatments in terms of leaf area is even more evident. Plants raised in the substrate amended with 25 nm *n*CeO_2_ develop a greater leaf area than the control (+18%, 261 vs. 221 cm^2^ plant^−1^), while treatment with *n*CeO_2_ 50 nm promoted a 44% increase (317 cm^2^ plant^−1^) compared to control (Figure 3C). Finally, according to the increase of leaf area, the leaves dry mass responded to the treatment. The ANOVA indicated a statistically significant effect of the factor “treatment” (*p* = 0.0482 *) and the post-hoc test showed that *n*CeO_2_ 25 nm and 50 nm stimulated a higher biomass production in leaves than control plants (1210 mg kg^−1^), with an increase of respectively 17% (1420 mg kg^−1^) and 24% (1511 mg kg^−1^). However, analyzing separately the results of the two species, it can be verified that data variability hides the effect of the size of *n*CeO_2_, which is not statistically significant for both *H. lanatus* and *D. tenuifolia* compared to control (Figure 3D).

**Figure 4:**
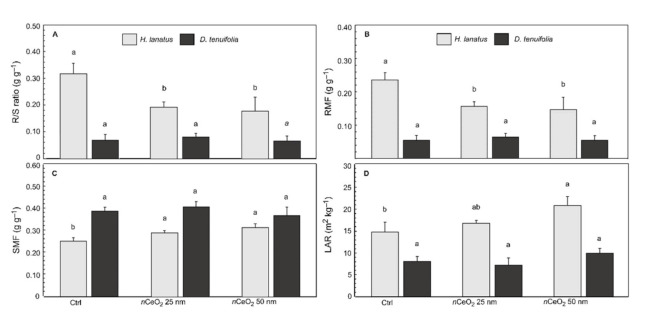
(**A**) Root to shoot ratio (R/S ratio), (**B**) Root mass fraction (RMF), (**C**) Stem mass fraction (SMF) and (**D**) Leaf area ratio (LAR) ± standard deviation of *H. lanatus* and *D. tenuifolia*. Comparison between control and plants grown in presence of 200 mg kg^−1^ *n*CeO_2_ having respectively 25 nm and 50 nm. For each species the statistically significant difference (*p* ≤ 0.05) between treatments is indicated by the letters according the Tukey’s test. −1

The biomass allocation in plants was affected by *n*CeO_2_ treatments, as well. Data analysis for these variables followed the same approach used for the biometric variables (Table S3). As happened for the measured variables, the effect of the factor “species” was always statistically significant at 2-way ANOVA, whereas the “treatment” was statistically significant for R/S ratio (*p*= 0.0038 **), RMF (*p* = 0.0070 **), and LAR (*p* = 0.0021 **) (Table S4). In this case, we deepened the data analysis with particular attention to the effect of *n*CeO_2_ size. As expected, taking into account the biometric data, the ratios that describe the biomass allocation in the fractions of *D. tenuifolia* did not respond to the treatments. In fact, for each parameter, the treatment with 25 nm and 50 nm *n*CeO_2_ did not produce statistically significant effects compared to the control plants (Figure 4A–D). Oppositely, in *H. lanatus* we observed statistically significant differences between treated and untreated plants. In addition, 2-way ANOVA showed a significant interaction “species” × “treatment” respectively in R/S ratio and RMF. According to the measured variables, statistics carried out for each species indicated that *H. lanatus* always responded to the *n*CeO_2_ treatments.

R/S ratio—and its inverse—are frequently used for an early assessment on the distribution of plant biomass. Data indicate that *n*CeO_2_ treated *H. lanatus* suffered a reduction of about 40% of the R/S ratio (*p* = 0.0085 **) compared to the control plants (Figure 4A), confirming the previously observed *n*CeO_2_ stimulating action on aerial biomass. The corresponding reduction in the allocation of biomass in the root system of *H. lanatus* is indicated in Figure 4B, where RMF shows a statistically significant reduction with respect control (−35.7%, *p* = 0.0105 *). In both cases, no statistically significant differences were observed in response to the different sizes of *n*CeO_2_ (Figure 4A–B).

In the case of SMF, the statistical analysis did not detect significant effects of the treatment on the accumulation of biomass in the drums (*p* = 0.1022 ns). This data contradicts the data on the increase in the number of stems in *H. lanatus* plants observed previously (Figure 4B). This is a consequence of the significant difference in the vegetative habitus of monocots compared to dicots. However, in the average of the species, the ANOVA did not reveal the expected effects on the SMF. Once again, after isolating the two species, the stimulating effect of *n*CeO_2_ in *H. lanatus* was highlighted (*p* = 0.0054 **) as it can be appreciated in Figure 4C. Therefore, being the increase of the stem biomass in treated plants statistically significant compared to the control ones (+ 17% averaging the Ce particle size), we can confirm the previous data.

The LAR also detected the effects of *n*CeO_2_ on plant canopy. In broad terms, this rate’s meaning can be interpreted as the plant’s investment of biomass in photosynthetic tissues to enhance light interception and carbon fixation. Since “the more a plant invests in leaf area, the higher the total carbon gain and the faster growth will be”, we found an increase in LAR in response to the presence of *n*CeO_2_ (*p* = 0.0178 *). More interesting is the fact that *H. lanatus* responded differently to the treatments. Compared to the control (14.8 m^2^ kg^−1^), we observed an increase of 12.8% (16.7 m^2^ kg^−1^) and 40% (20.8 m^2^ kg^−1^), respectively, for *n*CeO_2_ 25 nm and 50 nm (Figure 4D).

### 2.4. Ce in Plant Fractions

Table 1 presents the Ce concentration in different plant tissues. Statistical analysis showed a significant effect of the factor “species” (*p* = 0.0289 *) (Table S5). Hence, Ce assimilation is also very likely to occur in different ways in *H. lanatus* and *D. tenuifolia*. The Ce concentration measured in the plant roots is relatively low compared to the Ce concentration detected in the substrate after the soil treatment with *n*CeO_2_ (290 mg kg^−1^). Considering that the roots were acid washed to remove the fraction attached to the roots’ surface, Ce internalization was relatively low (range 3.04–16.1 mg kg^−1^).

Finally, it should be underlined that post-hoc test evidenced a statistically significant difference between Ce concentration in roots. In particular, in both species, the highest Ce concentration was observed in the plants’ roots treated with the smaller *n*CeO_2_ (Table 1).

The Ce translocation towards the aerial part of the plants was not very high. Compared to Ce accumulation in plant roots, data suggest that this element’s mobilization within plants had a different sense. Furthermore, plant species behaved differently. In *H. lanatus*, Ce concentration in stems and leaves was not statistically significant at ANOVA, whereas in *D. tenuifolia* they were (*p* = 0.0005 *** and *p* = 0.0006 ***, respectively). In sharp contrast to what was observed in roots, the highest Ce concentration was recorded in *n*CeO_2_ 50 nm treatment (Table 1).

### 2.5. Macro and Micronutrient Plant Uptake

The concentrations of essential macroelements (K, Mg, and P) and microelements (Cu, Fe, Mn, and Zn) in the tissues of *H. lanatus* and *D. tenuifolia* at maturity are respectively shown in Table 2 and Table 3: Tables S6–S8 reports the results of the statistical analysis. The monocot-dicot comparison was expected to highlight significant differences in the behaviour of the species. As previously observed, also in this case, the ANOVA showed that in the average of the treatments, the effect of the species factor was always statistically significant (*p* ≤ 0.001).

In the root tissues of *D. tenuifolia* the concentration of K, Mg and P was respectively 2.44, 2.71, and 5 times higher than *H. lanatus*. In the case of Na, the situation was the opposite and the greatest concentration of this element was recorded in the roots of *H. lanatus* (+1.67 times compared to *D. tenuifolia*, *p* ≤ 0.01) (Table 2). More important concerning the purpose of this study was the effect of the treatments. It was statistically significant only in the roots of *H. lanatus* for Mg and Na. The variation in the concentration of the two elements had an opposite trend. The concentration of Mg decreased (*p* = 0.0472 *) from 633 mg kg^−1^ (Ctrl) to 490 mg kg^−1^ (*n*CeO_2_ 50 nm), while Na concentration increased (*p* = 0.0458 *) from 331 mg kg^−1^ (Ctrl) to 686 mg kg^−1^ (*n*CeO_2_ 50 nm) (Table 2).

By observing the distribution of macronutrients in the aerial tissues of plants, a statistically significant effect of the factor is confirmed, especially in the case of K, Na and P. As expected, the highest concentration of K was found in *H. lanatus* (*p* = 0.0004 ***), while the greater accumulation of Na (*p* = 0.0009 ***) and *p* (*p* = 0.0198) was observed in the stems of *D. tenuifolia*. Moreover, the foliar tissues of *H. lanatus* accumulate more K than *D. tenuifolia* (*p* = 0.0115 *), while the dicot species allocates most Mg (*p* = 0.0000 ***). In both species, the aerial fractions do not show statistically significant effects due to the treatments with *n*CeO_2_ (Table 2). The statistically significant effect of the factor “species” was confirmed in the case of microelements. In particular, in the roots of *H. lanatus* the concentrations of Cu, Fe, Mn, and Zn are always higher than in *D. tenuifolia* (*p* = 0.0000 *** for each element) (Table S6). The same evidence is also confirmed in the stems for Cu (*p* = 0.0008 ***), Fe (*p* = 0.0289 *), Mn (*p* = 0.0000 ***) and Zn (*p* = 0.0108 *) (Table S7). Lastly, in the leaf tissues, we observed a higher concentration of Mn in *H. lanatus* (*p* = 0.0000 ***), while Zn accumulates more in the leaves of *D. tenuifolia* (*p* = 0.0000 ***) (Table S8).

Except Cu, the root uptake of micronutrients responded to the *n*CeO_2_ treatments, which determined a statistically significant response to Mn (*p*= 0.0441 *) and Zn (*p*= 0.0034 **) but in both species, no clear trends were recorded. On the contrary, we found an apparent adverse effect for Fe root uptake (*p* = 0.0375). In *H. lanatus*, the plant responds differently to *n*CeO_2_ 25 nm and *n*CeO_2_ 50 nm, which reduces the concentration of Fe of 25% and 56%, respectively. In *D. tenuifolia*, the response to *n*CeO_2_ 25 nm and 50 nm is not statistically different. However, treatment determined a substantial decrease in Fe accumulation (−70.5%) (Table 3). Finally, considering the leaf tissues, only the concentration of Cu in *D. tenuifolia* is affected by the treatments, which determines an increase in the concentration of the element (Table 3).

## 3. Discussion

This paper demonstrates that the growth of *D. tenuifolia* was not influenced by *n*CeO_2_, while, although *n*CeO_2_ concentration was not very high (200 mg kg^−1^) compared to several studies among those cited above, *H. lanatus* responded to the treatment.

The most evident effect of the treatments occurred on developing the aerial biomass of *H. lanatus*. This was unexpectedly observed in response to treatment with the *n*CeO_2_ 50 nm. As presented above, the larger nanoparticles stimulated a more significant number of stems per plant. In turn, greater tillering has increased aerial biomass accumulation and, therefore, a greater surface area of photosynthetic tissues was produced. This stimulating effect agrees with other observations made on autumn-spring cereals *T. aestivum* and *H. vulgare* [18,20]. In these experiments, plants were grown in soil amended with *n*CeO_2_ and responded to the highest treatment (500 mg kg^−1^) with increased plant height and aboveground dry biomass. In addition, the yield components of both species were negatively affected by the treatments. A confirmation of the negative impact of *n*CeO_2_ (at 800 mg kg^−1^) on grain yield was provided for *Zea mays*, as well [19].

Going back to the effects detected on *H. lanatus*, it must be specified that the observed increase in leaf area did not correspond the enhancement of the dry weight of the leaves as it would have been reasonable to expect. So, the newly formed leaves were thinner than those previously formed. According to similar observations on *H. vulgare* [21], it is likely that treated plants had a longer vegetative period than the controls. Conflicting evidence has been reported on dicotyledonous species exposed to *n*CeO_2_. Positive responses in terms of growth and yield of *L. esculentum* were recorded at low concentrations (10–125 mg kg^−1^) [27] whereas, at higher concentrations, *n*CeO_2_ adversely affect the plant metabolism and growth [38]. The same consequences have been verified in *G. max* [22], *C. sativus* [23] and *L. esculentum* [28].

Like other metal-oxide nanoparticles (e.g., *n*CuO, *n*TiO2, and *n*ZnO), *n*CeO_2_ has high stability and a low dissolution rate [38]. However, after interactions with plant tissues, nanoparticles may undergo dissolution and aggregation [39]. Aggregation is how the metal nanoparticles lose the monodisperse state and tend to associate in clusters of varying sizes. The process is mainly due to Van der Waals and electrostatic forces in a stock solution depending on the particle size, the surface properties, and charge [40]. The aggregation processes between nanoparticles can also occur in the soil, in the rhizosphere, and within plant tissues. However, the mechanisms of interaction between nanomaterials, possibly endowed with positive or negative charges, and biomolecules have not yet been fully elucidated [41].

The presence of aggregates of *n*CeO_2_ in plant tissues has been previously observed [42,43]. However, it was reported that the particle agglomeration occurs before the passage from roots to the other plant fractions [44]. In this experiment, the increase in the dissolved Ce fraction in roots exposed to *n*CeO_2_ 50 nm could be a proxy of an aggregation process that previously occurred within the root tissues. The aggregation of *n*CeO_2_ taken up by roots and the subsequent release of cerium ions inside roots prevents the translocation of *n*CeO_2_ towards the aerial plant biomass [45,46]. According to [47,48,49], fewer and smaller in size *n*CeO_2_ have detected the plant leaves. As for particle translocation, it has not conclusively clarified whether *n*CeO_2_ can move via a symplastic or apoplastic pathway [50].

Albeit such a specific aspect was not investigated in this study, the differences we observed can likely be explained at this level. So, we hypothesize that in the rhizosphere of *D. tenuifolia*, much more favorable conditions for the *n*CeO_2_ aggregation exist than in *H. lanatus*. On the other hand, the root morphology of monocots and dicots is very different [51]. The root uptake of nanoparticles is influenced by the specific surface of the root apparatus and the emissions of mucilage compounds and root exudates [44,52].

As for Ce concentration in plant fractions, an increase of Ce concentration in the roots with a linear trend was observed in treated plants while a limited translocation of Ce to the aboveground plant biomass. Our data corroborated those provided by several studies carried out in similar conditions [18,19,20,21,23,27,28].

Although *n*CeO_2_ treatments did not affect plant roots’ development, given their functional role, it is also essential to verify whether treatments influenced nutrient assimilation. Cerium belongs to the rare earth elements (REE). Due to the increasing industrial exploitation of such elements, they are currently considered emerging pollutants. Their plant toxicity mechanisms are not completely clear yet. However, a solid demonstration regarding the influence of Ce on plant nutrition was already provided [53,54].

The elemental composition of edible products of crops grown in a solid media treated with *n*CeO_2_ has been previously investigated in monocots [19,20,55,56,57] and dicots [27,58,59,60]. These studies evaluated the nutritional value of crop yield eventually influenced by the ENMs. The focus of this work is mainly ecological. However, even in this perspective, it is relevant to check whether plant exposure to *n*CeO_2_ has any consequences on the uptake of macro and microelements, which can be evidence of plant stress and negative consequences on the food web [61].

Considering literature data, we can draw the following summary considerations. First, the concentration of macro and micronutrients in plants varies with soil types; therefore, even from solidly organized experiments, it is not easy to generalize nutritional imbalances in plants exposed to ENM. Second, *n*CeO_2_ influence the assimilation of some elements in the exposed plants. As for macronutrients, the literature provides clear indications of the negative effect of *n*CeO_2_ on the uptake of some elements. Data on *H. lanatus* confirm a significant reduction in Mg uptake due to *n*CeO_2_, already observed in *T. aestivum* [57,60]. As for Na, it was reported a decrease of concentration in barley plants, while we observed an increase of this element in treated *H. lanatus*. Still conflicting indications concern the assimilation of K and P in plants treated with *n*CeO_2_ [20,60], whereas no responses were observed in *H. lanatus* and *D. tenuifolia*.

The root uptake of micronutrients is more sensitive to the presence of *n*CeO_2_ than macronutrients. Therefore, a micronutrient imbalance could be the most critical effect of ENMs on plants. In this study, the clearest example of the influence of *n*CeO_2_ on the assimilation of nutrients concerned Fe. A relevant reduction in root Fe uptake in both *H. lanatus* and *D. tenuifolia* was observed. Moreover, in the dicot, the lower Fe accumulation was observed in the plant stems but not in the leaves. Contrarily, other authors reported a remarkable increase in Fe accumulation in soybean plants [60]. Manganese uptake in *H. lanatus* and *D. tenuifolia* was negatively affected by *n*CeO_2_ 25 nm and, to a lesser extent, by the larger *n*CeO_2_. Similar results were obtained on *Triticum aestivum* [57] but not on *Glycine max* [22]. Finally, the Zn root concentration in treated plants compared to the control decreased in *H. lanatus* and increased in *D. tenuifolia*. Similar contradictory results are provided by the literature [20,60].

## 4. Materials and Methods

### 4.1. nCeO_2_ Characterization

The cerium oxide nanopowders (*n*CeO_2_) with an average particle size of 25 nm and 50 nm respectively were purchased from Sigma-Aldrich (St. Louis, MO, USA). *n*CeO_2_ have a density of 7.13 g mL^−1^ at 25 °C and 99.95% of purity (81.25% of Ce). The suspensions were characterized for hydrodynamic diameter (Hd), Z–average size, relative polydispersity index (PDI), and ζ–potentials. The size and average shape were measured with a transmission electron microscope (TEM, FEI Tecnai F20, FEI Company, Eindhoven, The Netherlands). The *n*CeO_2_ were suspended in deionized water and sonicated in a water bath for 60 min with a sonication intensity of 180 W cm^−2^. The hydrodynamic diameter (Hd) and the Z–average size on *n*CeO_2_ were measured by dynamic light scattering (DLS) on a Zetasizer Nano ZS (Malvern Ltd., Worcestershire, UK). ζ–potentials were quantified by laser Doppler velocimetry as the electrophoretic mobility, using a disposable electrophoretic cell (DTS1061, Malvern Ltd., Worcestershire, UK).

### 4.2. Experimental Conditions

Seeds of *H. lanatus* were purchased by SemeNostrum (Udine, Italy), while seeds of *D. tenuifolia* were provided by Sementi Bruni (Corbetta, Milan, Italy). The soil used for this experiment was Compo Sana organic potting mix (soil pH = 6.8–7.2). The potting substrates were amended with two water suspensions, one with 25 nm *n*CeO_2_ and the other with 50 nm *n*CeO_2_, in order to reach a final substrate concentration of 200 mg kg^−1^ *n*CeO_2_. Before this amendment, which occurred in a single dose through irrigation, the suspensions were stirred and sonicated for 30 min to avoid aggregation. Tap water was used as control. After the irrigation, the amended soils were carefully mixed in order to obtain uniformity. Subsequently, the *n*CeO_2_ amended substrates were stored in the dark at 15 °C for three days for conditioning before planting seeds. After equilibration, 500 g of control and amended substrate were put in the pots (Figure S1). The experiment was carried out in a semi-controlled greenhouse facility.

Seeds were sowed about 2.5 cm deep in the soil and pots were placed in full sunlight, at 18–27 °C (night/day) with a relative humidity around 60% (Figure S6). Two weeks after sowing, the seedlings were thinned to two seedlings per pot. An additional set of plants were grown to observe the entry of *n*CeO_2_ into plant tissues. During the trial, pots were irrigated every three days and randomly reallocated every week. During the plant growth the pots were irrigated to maintain the substrate at 60% of water holding capacity (Figures S7 and S8). After 60 days, control and treated plants were harvested. Fresh plant biomass was separated in roots, stems, and leaves and weighed. Then the plant fractions were thoroughly washed in tap water and rinsed three times with distilled water. In addition, roots were washed in 400 mL of 0.01 M of HNO_3_ in a shaker bath at 300 rpm for 5 min to remove metal ions adsorbed at the surface. Leaf area was measured using a LI-3100C Area Meter (Li-Cor Corporation, Lincoln, NE, USA). Finally, the plant fractions were oven dried at 60 °C for three days and weighed. Additional information regarding the experimental setup are reported in Supplementary Information.

### 4.3. Detection of nCeO_2_ in Plant Fractions

Before setting up the main factorial experiment, it was carried out a preliminary test with the aim of verifying that in our experimental conditions it would have been possible to trace the *n*CeO_2_ inside the plant tissues. For this reason, other plants were grown under the same conditions as in the main experiment. Twenty days after germination, the uptake and translocation of *n*CeO_2_ in plants was investigated by enzymatic digestion [62]. The digesting enzyme used is Macerozyme R–10 Pectinase from *Rhizopus* sp. (Sigma Aldrich, St. Louis, MO, USA). The technical details are reported in Supplementary Information.

### 4.4. Cerium Concentration in Plant Tissues

An amount of 0.3 g was sampled from the oven-dried plant fraction and digested on a CEM microwave oven (MARS Xpress, CEM, Matthews, NC, USA), using 9 mL of HNO_3_ (65%) and 1 mL of hydrogen peroxide (H_2_O_2_) in Teflon cylinders at 180 °C, according to the USEPA 3052 method [63] After mineralization, plant extracts were filtered at room temperature under fume hood with Whatman 0.45 μm PTFE membrane filters, and finally diluted and analyzed. Determinations of the Ce total content were carried out using a ICP-MS NexION 350 (PerkinElmer, Waltham, MA, USA). The accuracy of the analytical procedure adopted for ICP-MS analysis was checked by running standard solutions every 20 samples. Yttrium was used as the internal standard.

### 4.5. Calculations and Statistical Analysis

The trial was set up in a randomized experimental design with the following factors: (i) two plant species, (ii) Ce concentration: 0 (Ctrl) and 200 mg kg^−1^ *n*CeO_2_, (iii) *n*CeO_2_ particle size: 25 nm and 50 nm, respectively. Each treatment was replicated four times. From the dry weight of plant fractions and leaf area, the following ratios were calculated: root to shoot ratio (R/S ratio), the fraction of total plant biomass allocated to roots (RMF, root mass fraction), and stems (SMF, stem mass fraction), and the leaf area ratio (LAR) (Table S3). Statistical analysis was carried out with one-, two- and three-way ANOVA. When necessary, data were subjected to logarithmic transformation prior to analysis, which effectively homogenized the variances and produced approximately normal distributions. Statistically significant differences correspond to *p* ≤ 0.05. In case of significant effects, Tukey’s Multiple Comparison test was used to analyze individual effects. Different letters in tables and graphics are used to indicate means that are statistically different. Single particle inductively coupled plasma mass spectrometry (sp-ICP-MS) data on *n*CeO_2_ size distribution were processed by means of Syngistix Nano Application Module software (Perkin Elmer, Waltham, MA, USA), and interpolated with Gaussian curves.

## 5. Conclusions

Literature shows that the most studied terrestrial plant species are radish, rape, ryegrass, lettuce, corn, and cucumber. However, the documented and modeled flows of ENMs in the environment also affect wild plants. Such plants live in uncultivated natural areas and contribute to supporting the food web and ecosystem services (e.g., C-cycle, interactions with microbes and insects).

In this study, the first question concerned the uptake and translocation of *n*CeO_2_ within the studied plant species. Plant roots indeed assimilated *n*CeO_2_ with a magnitude depending on their size; however, only a small fraction of them moved through to the aerial plant parts. A difference in *n*CeO_2_ assimilation due to the species was observed, as well. A second aspect we studied concerns the effects on plant growth. During the growth cycle, the monocot species benefited from the treatment with *n*CeO_2_ 50 nm by accumulating more biomass in the aerial fractions and increasing the photosynthesizing surface. However, it must be emphasized that the concentration of *n*CeO_2_ we used is not very high, being comprised—according to the literature—in the range that stimulates plant growth. This likely hidden the expected *n*CeO_2_ size-effects, whereas, at higher concentrations, we might have appreciated this effect. However, even in the absence of evident negative anomalies on plant growth, we observed alterations in macro and micronutrients’ root uptake.

Whether early studies on ENMs’ plant relationships regarded punctual observations on plants exposed to high doses of ENMs, current investigations are currently developing more and more intensely in different directions. Some examples are (i) effects of ENMs low doses over time, (ii) ENMs biotransformation, and (iii) the omics data describing the complex plant molecular networks in ENMs response. However, from an ecological perspective, it is of fundamental importance to develop adequate studies on spontaneous species. However, the impact of these materials on food chains has not yet been adequately studied.

In general, wild plant species have high plasticity by combining both the long-term accumulation of genetic changes and conserving survival strategies through time. On the other hand, the requested high productive performances of crops are often genetically obtained to the detriment of biotic and abiotic stresses’ adaptation ability. So, it seems plausible to hypothesize that the ENMs-plants relationships may be different in wild species than cultivated ones, also keeping into account that they are possibly exposed to ENMs fluxes for a more extended time. These aspects deserve adequate attention and will undoubtedly open new investigation fields in understanding the consequences ENMs fluxes in the ecosystems.

## Figures and Tables

**Table 1:** Ce concentration (µg kg^−1^ dry weight) in roots, stems and leaves of *H. lanatus* and *D. tenuifolia*. Values are means ± standard deviation (SD). Between treatments means with the same letter are not significantly different at Tukey’s test (*p* ≤ 0.05). In mean comparison between species, ns is not significant at *p* ≤ 0.05, *, indicate significance at *p* ≤ 0.05.

Treatment	Ce Roots		Ce Stems		Ce Leaves	
	*H. lanatus*	*D. tenuifolia*	*H. lanatus*	*D. tenuifolia*	*H. lanatus*	*D. tenuifolia*
Ctrl	822 ± 241c	305 ± 197c	456 ± 127a	225 ± 180b	625 ± 231a	145 ± 158b
nCeO2 25 nm	16,079 ± 5601a	9392 ± 2797a	556 ± 257a	140 ± 55.7b	165 ± 191a	99.6 ± 129b
nCeO2 50 nm	5273 ± 1926b	3037 ± 666b	661 ± 472a	886 ± 85.4a	496 ± 298a	1044 ± 195a
Mean	7391 ± 7413	4244 ± 4287 *	558 ±290	417 ± 369 ns	429 ± 279	430 ± 482ns

**Table 2:** Macronutrient concentration (mg kg^−1^ dry weight) in roots, stems and leaves of *H. lanatus* and *D. tenuifolia*. Values are means ± standard deviation (SD). Between treatments means with the same letter are not significantly different at Tukey’s test (*p* ≤ 0.05). In mean comparison between species, ns is not significant at *p* ≤ 0.05, *, and *** indicate significance at *p* ≤ 0.05, and *p* ≤ 0.001.

Treatment	K		Mg		Na		P	
	*H. lanatus*	*D. tenuifolia*	*H. lanatus*	*D. tenuifolia*	*H. lanatus*	*D. tenuifolia*	*H. lanatus*	*D. tenuifolia*
	Roots							
Ctrl	5500 ± 657a	14,777 ± 743a	633 ± 81.8a	1302 ± 87.4a	331 ± 79.8b	187 ± 98.7a	1224 ± 136a	6669 ± 840a
*n*CeO_2_ 25 nm	6869 ± 1352a	13,288 ± 2805a	588 ± 35.4ab	1551 ± 334a	390 ± 125ab	249 ± 123a	1465 ± 125a	5969 ± 1487a
*n*CeO_2_ 50 nm	5881 ± 791a	16,545 ± 2015a	490 ± 33.5b	1780 ± 355a	686 ± 191a	406 ± 100a	1363 ± 108a	7659 ± 742 a
Mean	6084 ± 1047	14,870 ± 2413 ***	570 ± 79.2	1544 ± 337 ***	469 ± 204	281 ± 129 *	1351 ± 150	6766 ± 1230 ***
	Stems							
Ctrl	28,927 ± 2252a	23,694 ± 2802a	1032 ± 110a	1014 ± 394a	115 ± 11.7ab	265 ± 142a	2836 ± 287a	3431 ± 737a
*n*CeO_2_ 25 nm	31,580 ± 2978a	22,466 ± 3856a	1114 ± 10.4a	878 ± 277a	95.5 ± 9.12b	318 ± 29.6a	2524 ± 167a	2875 ± 9.76a
*n*CeO_2_ 50 nm	32,065 ± 3806a	26,355 ± 663a	1117 ± 37.7a	932 ± 208a	128 ± 15.2a	519 ± 267a	2668 ± 291a	3402 ± 661a
Mean	30,857 ± 3041	24,172 ± 2964 ***	1088 ± 71.9	941 ± 194ns	113 ± 17.8	368 ± 176 ***	2676 ± 259	3234 ± 437 *
	Leaves							
Ctrl	31,314 ± 2192a	30,115 ± 4571a	1547 ± 80.9a	2312 ± 313a	150 ± 46.6a	1249 ± 325a	3671 ± 462a	3138 ± 628a
*n*CeO_2_ 25 nm	32,772 ± 2096a	22,672 ± 3176a	1397 ± 51.9a	2459 ± 94.6a	81 ± 16.8a	803 ± 219a	3335 ± 401a	2677 ± 429a
*n*CeO_2_ 50 nm	31,610 ± 1854a	30,057 ± 3631a	1508 ± 13.2a	2267 ± 229a	126 ± 77.6a	1094 ± 206a	3163 ± 473a	3719 ± 388a
Mean	31,899 ± 1836	27,615 ± 5106 *	1455 ± 68.2	2346 ± 192 ***	119 ± 55.1	1049 ± 296 ***	3390 ± 447	3178 ± 664ns

**Table 3:** Macronutrient concentration (mg kg^−1^ dry weight) in roots, stems and leaves of *H. lanatus* and *D. tenuifolia*. Values are means ± standard deviation (SD). Between treatments means with the same letter are not significantly different at Tukey’s test (*p* ≤ 0.05). In mean comparison between species, ns is not significant at *p* ≤ 0.05, *, and *** indicate significance at *p* ≤ 0.05, and *p* ≤ 0.001, respectively.

Treatment	K		Mg		Na		P	
	*H. lanatus*	*D. tenuifolia*	*H. lanatus*	*D. tenuifolia*	*H. lanatus*	*D. tenuifolia*	*H. lanatus*	*D. tenuifolia*
	Roots							
Ctrl	7.35 ± 0.27a	7.35 ± 0.27a	138 ± 38.3a	46.3 ± 3.14a	19.1 ± 3.34a	4.21 ± 0.86a	159 ± 15.9a	40.3 ± 5.11b
*n*CeO_2_ 25 nm	7.84 ± 2.06a	7.84 ± 2.06a	103 ± 20.9ab	14.3 ± 1.97b	12.3 ± 13.6b	2.59 ± 0.44b	101 ± 13.7b	68 ± 10.1a
*n*CeO_2_ 50 nm	7.35 ± 1.25a	7.35 ± 1.25a	60.7 ± 19b	13 ± 3.28b	16.7 ± 2.12ab	4.15 ± 0.51ab	131 ± 0.24ab	53.9 ± 5.38ab
Mean	7.51 ± 1.24	7.51 ± 1.24	101 ± 41.2	24.5 ± 14.9 ***	16 ± 3.74	3.65 ± 1.03 ***	130 ±27.3	54 ± 13.6 ***
	Stems							
Ctrl	2.99 ± 0.66a	2.99 ± 0.66a	30.2 ± 11.3a	23.4 ± 10.3a	69.3 ± 19.3a	4.04 ± 0.98a	51.5 ± 4.55a	30 ± 10.6a
*n*CeO_2_ 25 nm	2.97 ± 0.37a	2.97 ± 0.37a	17.7 ± 3.65a	14.3 ± 5.11ab	31.4 ± 11.4a	3.15 ± 0.25a	47.0 ± 3.99a	29.5 ± 8.12a
*n*CeO_2_ 50 nm	2.76 ± 0.61a	2.76 ± 0.61a	18.7 ± 1.89a	4.97 ± 1.86b	49.9 ± 15.8a	4.71 ± 0.83a	41.0 ± 11.3a	30.9 ± 17.3a
Mean	2.91± 0.5	2.91± 0.5	22.2 ± 8.53	14.2 ± 10.4*	51.1 ± 20.5	3.94 ± 0.92 ***	46.5 ± 7.85	31.8 ± 10.3 *
	Leaves							
Ctrl	3.56 ± 0.52a	3.56 ± 0.52a	34.5 ± 4.60a	42.5 ± 4.52a	47.3 ± 16.9a	9.96 ± 0.80a	26.8 ± 0.40a	100 ± 26.2a
*n*CeO_2_ 25 nm	4.47 ± 0.48a	4.47 ± 0.48a	36.1 ± 3.54a	28.9 ± 5.26a	26.8 ± 9.11a	10.7 ± 0.74a	29.2 ± 6.29a	96.5 ± 22.5a
*n*CeO_2_ 50 nm	4.13 ± 0.57a	4.13 ± 0.57a	34.0 ± 2.76a	36.0 ± 6.49a	37.6 ± 8.72a	13.5 ± 4.44a	26.0 ± 5.23a	70.0 ± 22.2a
Mean	4.06 ± 0.6	4.06 ± 0.6	34.9 ± 3.35	35.8 ± 7.29ns	37.3 ±13.8	11.4 ±2.98 ***	27.3 ± 4.34	88.4 ± 20.9 ***

## Data Availability

The data presented in this study are available on request from the corresponding author.

## Supplementary Materials

The following are available online at https://www.mdpi.com/2223-7747/10/2/335/s1, Figure S1: Preparation of the experiment: filling the pots with the *n*CeO_2_ amended substrate. Figure S2: Plantlets of *H. lanatus* and *D. tenuifolia* 10 d after sowing. Figure S3: Plants of *H. lanatus* and *D. tenuifolia* 30 day after sowing. Figure S4: Plants of *H. lanatus* (in the background) and *D. tenuifolia* before biomass harvesting. Figure S5: Stems dry matter ± standard deviation of *H. lanatus* and *D. tenuifolia*. Comparison between control and plants grown in presence of 200 mg kg^−1^ *n*CeO_2_ having respectively 25 nm and 50 nm. For each species the statistically significant difference (*p* ≤ 0.05) between treatments is indicated by the letters using one-way ANOVA followed by Tukey’s test. Figure S6: Total plant dry matter ± standard deviation of *H. lanatus* and *D. tenuifolia*. Comparison between control and plants grown in presence of 200 mg kg^−1^ *n*CeO_2_ having respectively 25 nm and 50 nm. For each species the statistically significant difference (*p* ≤ 0.05) between treatments is indicated by the letters using one-way ANOVA followed by Tukey’s test. Figure S7: Leaf mass fraction ± standard deviation of *H. lanatus* and *D. tenuifolia*. Comparison between control and plants grown in presence of 200 mg kg^−1^ *n*CeO_2_ having respectively 25 nm and 50 nm. For each species the statistically significant difference (*p* ≤ 0.05) between treatments is indicated by the letters using one-way ANOVA followed by Tukey’s test. Figure S8: Specific leaf area* ± standard deviation of *H. lanatus* and *D. tenuifolia*. Comparison between control and plants grown in presence of 200 mg kg^−1^ *n*CeO_2_ having respectively 25 nm and 50 nm. For each species the statistically significant difference (*p* ≤ 0.05) between treatments is indicated by the letters using one-way ANOVA followed by Tukey’s test. * According to Evans (1972) SLA is the total leaf area of a plant divided by the total leaf weight. This ratio has a relevant ecological importance as describes the allocation of leaf biomass relative to leaf area which in turns refers to carbon gain relative to water loss, within a plant canopy (Gunn et al., 1999). Table S1—Most frequent particle size, mean particle size, number of peaks and content of dissolved Ce determined by sp–ICP–MS analysis after enzymatic extraction on roots and leaves of *H. lanatus* and *D. tenuifolia* treated with *n*CeO_2_ 200 mg L^−1^. Table S2—Two-way ANOVA *p* value determined for biometric variables of *H. lanatus* and *D. tenuifolia*. ns is not significant at *p* ≤ 0.05, *, ** and *** indicate significance at *p* ≤ 0.05, *p* ≤ 0.01 and *p* ≤ 0.001, respectively. Table S3—Biomass allocation variables calculated from plant measurements (Porter et al., 2011). Table S4—Two-way ANOVA *p* value determined for biometric ratios calculated for *H. lanatus* and *D. tenuifolia*. ns is not significant at *p* ≤ 0.05, *, ** and *** indicate significance at *p* ≤ 0.05, *p* ≤ 0.01 and *p* ≤ 0.001, respectively. Table S5—Two-way ANOVA *p* value determined for Ce concentration in plant fractions of *H. lanatus* and *D. tenuifolia*. ns is not significant at *p* ≤ 0.05, *, ** and *** indicate significance at *p* ≤ 0.05, *p* ≤ 0.01 and *p* ≤ 0.001, respectively. Table S6—Two-way ANOVA *p* value for concentration of macronutrients and micronutrients in roots of *H. lanatus* and *D. tenuifolia*. ns is not significant at *p* ≤ 0.05, *, ** and *** indicate significance at *p* ≤ 0.05, *p* ≤ 0.01 and *p* ≤ 0.001, respectively. Table S7—Two-way ANOVA *p* value for concentration of macronutrients and micronutrients in stems of *H. lanatus* and *D. tenuifolia*. ns is not significant at *p* ≤ 0.05, *, ** and *** indicate significance at *p* ≤ 0.05, *p* ≤ 0.01 and *p* ≤ 0.001, respectively. Table S8—Two-way ANOVA *p* value for concentration of macronutrients and micronutrients in leaves of *H. lanatus* and *D. tenuifolia*. ns is not significant at *p* ≤ 0.05, *, ** and *** indicate significance at *p* ≤ 0.05, *p* ≤ 0.01 and *p* ≤ 0.001, respectively.

## References

[1] Bainbridge W.S., Roco M.C. (2016). Science and technology convergence: With emphasis for nanotechnology-inspired convergence. J. Nanopart. Res..

[2] Mortimer M., Holden P.A., Marmiroli N., White J.C., Song J. (2019). Fate of engineered nanomaterials in natural environments and impacts on ecosystems. Exposure to Engineered Nanomaterials in the Environment.

[3] Keller A.A., McFerran S., Lazareva A., Suh S. (2013). Global life cycle releases of engineered nanomaterials. J. Nanopart. Res..

[4] Holden P.A., Gardea-Torresdey J.L., Klaessig F., Turco R.F., Mortimer M., Hund-Rinke K., Cohen Hubal E.A., Avery D., Barceló D., Behra R. (2016). Considerations of environmentally relevant test conditions for improved evaluation of ecological hazards of engineered nanomaterials. Environ. Sci. Technol..

[5] Giese B., Klaessig F., Park B., Kaegi R., Steinfeldt M., Wigger H., von Gleich A., Gottschalk F. (2018). Risks, release and con-centrations of engineered nanomaterial in the environment. Sci. Rep..

[6] Sun T.Y., Bornhöft N.A., Hungerbühler K., Nowack B. (2016). Dynamic probabilistic modeling of environmental emissions of engineered nanomaterials. Environ. Sci. Technol..

[7] Piccinno F., Gottschalk F., Seeger S., Nowack B. (2012). Industrial production quantities and uses of ten engineered nanomaterials in Europe and the world. J. Nanopart. Res..

[8] Hamidat M., Barakat M., Ortet P., Chanéac C., Rose J., Bottero J.Y., Heulin T., Achouak W., Santaella C. (2016). Design defines the effects of nanoceria at a low dose on soil microbiota and the potentiation of impacts by the canola plant. Environ. Sci. Technol..

[9] Meesters J.A.J., Quik J.T.K., Koelmans A.A., Hendriks A.J., van de Meent D. (2016). Multimedia environmental fate and specia-tion of engineered nanoparticles: A probabilistic modelling approach. Environ. Sci. Nano.

[10] Cross R.K., Tyler C.R., Galloway T.S. (2019). 2019. The fate of cerium oxide nanoparticles in sediments and their routes of uptake in a freshwater worm. Nanotoxicology.

[11] Gottschalk F., Lassen C., Kjoelholt J., Christensen F., Nowack B. (2015). Modeling flows and concentrations of nine engineered nanomaterials in the Danish environment. Int. J. Environ. Res. Public. Health.

[12] Miralles P., Church T.L., Harris A.T. (2012). Toxicity, uptake, and translocation of engineered nanomaterials in vascular plants. Environ. Sci. Technol..

[13] Zhang P., Ma Y., Zhang Z., Siddiqui M., Al-Whaibi M., Mohammad F. (2015). Interactions between engineered nanomaterials and plants: Phytotoxicity, uptake, translocation, and biotransformation. Nanotechnology and Plant Sciences.

[14] Zuverza-Mena N., Martínez-Fernández D., Du W., Hernandez-Viezcas J.A., Bonilla-Bird N., López-Moreno L.M., Komárek M., Peralta-Videa J.R., Gardea-Torresdey J.L. (2017). Exposure of engineered nanomaterials to plants: Insights into the physiological and biochemical responses—A review. Plant Physiol. Biochem..

[15] Lowry G.V., Avellan A., Gilbertson L.M. (2019). Opportunities and challenges for nanotechnology in the agri-tech revolution. Nat. Nanotechnol..

[16] Marchiol L., Iafisco M., Fellet G., Adamiano A. (2020). Nanotechnology support the next agricultural revolution: Perspectives to enhancement of nutrient use efficiency. Adv. Agron..

[17] Lizzi D., Mattiello A., Marchiol L., Tripathi D.K., Ahmad P., Sharma S., Chauhan D. (2017). Impacts of cerium oxide nanoparticles (*n*CeO_2_) on crop plants: A concentric overview. Nanomaterials in Plants, Algae and Micro-Organisms. Concepts and Controversies.

[18] Rico C.M., Lee S.C., Rubenecia R., Mukherjee A., Hong J., Peralta-Videa J.R., Gardea-Torresdey J.L. (2014). Cerium oxide nanoparticles impact yield and modify nutritional parameters in wheat (*Triticum aestivum* L.). J. Agric. Food Chem..

[19] Zhao L., Sun Y., Hernandez-Viezcas J.A., Hong J., Majumdar S., Niu G., Duarte-Gardea M.O., Peralta-Videa J.R., Gardea-Torresdey J.L. (2015). Monitoring the environmental effects of CeO_2_ and ZnO nanoparticles through the life cycle of corn (*Zea mays*) plants and in situ µ-XRF mapping of nutrients in kernels. Environ. Sci. Technol..

[20] Rico C.M., Barrios A.C., Tan W., Rubenecia R., Lee S.C., Varela-Ramirez A., Peralta-Videa J.R., Gardea-Torresdey J.L. (2015). Physiological and biochemical response of soil-grown barley (*Hordeum vulgare* L.) to cerium oxide nanoparticles. Environ. Sci. Pollut. Res..

[21] Marchiol L., Mattiello A., Pošćić F., Fellet G., Zavalloni C., Carlino E., Musetti R. (2016). Changes in physiological and agronomical parameters of barley (*Hordeum vulgare*) exposed to cerium and titanium dioxide nanoparticles. Int. J. Environ. Res. Public Health.

[22] Priester J.H., Ge Y., Mielke R.E., Horst A.M., Moritz S.C., Espinosa K., Gelb J., Walker S.L., Nisbet R.M., An Y.L. (2012). Soybean susceptibility to manufactured nanomaterials with evidence for food quality and soil fertility interruption. Proc. Natl. Acad. Sci. USA.

[23] Zhao L., Sun Y., Hernandez-Viezcas J.A., Servin A.D., Hong J., Niu G., Peralta-Videa J.R., Duarte-Gardea M., Gardea-Torresdey J.L. (2013). Influence of CeO_2_ and ZnO nanoparticles on cucumber physiological markers and bioaccumulation of Ce and Zn: A life cycle study. J. Agric. Food Chem..

[24] Trujillo-Reyes J., Vilchis-Nestor A.R., Majumdar S., Peralta-Videa J.R., Gardea-Torresdey J.L. (2013). Citric acid modifies surface properties of commercial CeO_2_ nanoparticles reducing their toxicity and cerium uptake in radish (*Raphanus sativus*) seedlings. J. Hazard. Mater..

[25] Wang Q., Ma X., Zhang W., Peia H., Chen Y. (2012). The impact of cerium oxide nanoparticles on tomato (*Solanum lycopersicum* L.) and its implications for food safety. Metallomics.

[26] Vittori Antisari L., Carbone S., Gatti A., Vianello A., Nannipieri P. (2015). Uptake and translocation of metals and nutrients in tomato grown in soil polluted with metal oxide (CeO_2_, Fe_3_O_4_, SnO_2_, TiO_2_) or metallic (Ag, Co, Ni) engineered nanoparticles. Environ. Sci. Pollut. Res..

[27] Barrios A.C., Rico C.M., Trujillo-Reyes J., Medina-Velo I.A., Peralta-Videa J.R., Gardea-Torresdey J.L. (2016). Effects of uncoated and citric acid coated cerium oxide nanoparticles, bulk cerium oxide, cerium acetate, and citric acid on tomato plants. Sci. Total Environ..

[28] Barrios A.C., Medina-Velo I.A., Zuverza-Mena N., Dominguez O.E., Peralta-Videa J.R., Gardea-Torresdey J.L. (2017). Nutritional quality assessment of tomato fruits after exposure to uncoated and citric acid coated cerium oxide nanoparticles, bulk cerium oxide, cerium acetate and citric acid. Plant. Physiol. Biochem..

[29] Adisa O., Rawat S., Laxma V., Pullagurala R., Dimkpa C.O., Elmer W.H., White J.C., Hernandez-Viezcas J.A., Peralta-Videa J.R., Gardea-Torresdey J.L. (2020). Nutritional status of tomato (*Solanum lycopersicum*) fruit grown in *Fusarium* infested soil: Impact of cerium oxide nanoparticles. J. Agric. Food Chem..

[30] Antonelli A., Fry C., Smith R.J., Simmonds M.S.J., Kersey P.J., Pritchard H.W., Abbo M.S., Acedo C., Adams J., Ainsworth A.M. (2020). State of the World’s Plants and Fungi 2020.

[31] Reddy Pullagurala V.L., Adisa I.O., Rawat S., White J.C., Zuverza-Mena N., Hernandez-Viezcas J.A., Peralta-Videa J.R., Gardea-Torresdey J.L., Marmiroli N., White J.C., Song J. (2019). Fate of engineered nanomaterials in agroenvironments and impacts on agroecosystems. Exposure to Engineered Nanomaterials in the Environment.

[32] Ekperusia O., Sikokic F.D., Nwachukwud E.O. (2019). Application of common duckweed (*Lemna minor*) in phytoremediation of chemicals in the environment: State and future perspective. Chemosphere.

[33] Geitner N.K., Cooper J.L., Avellan A., Castellon B.T., Perrotta B.G., Bossa N., Simonin M., Anderson S.M., Inoue S., Hochella M.F. (2018). Size-based differential transport, uptake, and mass distribution of ceria (CeO_2_) nanoparticles in wetland mesocosms. Environ. Sci. Technol..

[34] Yin L., Colman B.P., McGill B.M., Wright J.P., Bernhardt E.S. (2012). Effects of silver nanoparticle exposure on germination and early growth of eleven wetland plants. PLoS ONE.

[35] Aleksandrowicz-Trzcińska M., Bederska-Błaszczyk M., Szaniawski A., Olchowik J., Studnicki M. (2019). The effects of copper and silver nanoparticles on container-grown Scots Pine (*Pinus sylvestris* L.) and Pedunculate Oak (*Quercus robur* L.) seedlings. Forests.

[36] Stace C. (1997). New Flora of the British Isles.

[37] Thompson J.D., Turkington R. (1988). The biology of Canadian weeds. 82. *Holcus lanatus* L.. Can. J. Plant. Sci..

[38] Tan W., Peralta-Videa J.R., Gardea-Torresdey J.L. (2018). Interaction of titanium dioxide nanoparticles with soil components and plants: Current knowledge and future research needs-a critical review. Environ. Sci. Nano.

[39] Keller A.A., Huang Y., Nelson J. (2018). Detection of nanoparticles in edible plant tissues exposed to nano-copper using single-particle ICP-MS. J. Nanopart. Res..

[40] Giorgi F., Coglitore D., Curran J.M., Gilliland D., Macko P., Whelan M., Worth A., Patterson E.A. (2019). The influence of inter-particle forces on diffusion at the nanoscale. Sci. Rep..

[41] Tian X., Chong Y., Ge C. (2020). Understanding the nano–bio interactions and the corresponding biological responses. Front. Chem..

[42] Spielman-Sun E., Avellan A., Bland G.D., Tappero R.V., Acerbo A.S., Unrine J.M., Giraldo J.P., Lowry G.V. (2019). Nanoparticle surface charge influences translocation and leaf distribution in vascular plants with contrasting anatomy. Environ. Sci. Nano.

[43] Zhang W., Dan Y., Shi H., Ma X. (2017). Elucidating the mechanisms for plant uptake and in-planta speciation of cerium in radish (*Raphanus sativus* L.) treated with cerium oxide nanoparticles. J. Environ. Chem. Eng..

[44] Avellan A., Schwab F., Masion A., Chaurand P., Borschneck D., Vidal V., Rose J., Santaella C., Levard C. (2017). Nanoparticle uptake in plants: Gold nanomaterial localized in roots of *Arabidopsis thaliana* by x-ray computed nanotomography and hyperspectral imaging. Environ. Sci. Technol..

[45] Slomberg D.L., Schoenfisch H.M. (2012). Silica nanoparticle phytotoxicity to *Arabidopsis thaliana*. Environ. Sci. Technol..

[46] Larue C., Laurette J., Herlin-Boime N., Khodja H., Fayard B., Flank A.M., Brisset F., Carriere M. (2012). Accumulation, translocation and impact of TiO2 nanoparticles in wheat (*Triticum aestivum* spp.): Influence of diameter and crystal phase. Sci. Total Environ..

[47] Kińska K., Jiménez-Lamana J., Kowalska J., Krasnodębska-Ostręga B., Szpunar J. (2018). Study of the uptake and bioaccumulation of palladium nanoparticles by *Sinapis alba* using single particle ICP-MS. Sci. Total Environ..

[48] Wojcieszek J., Jiménez-Lamana J., Bierła K., Ruzik L., Asztemborska M., Jarosz M., Szpunar J. (2019). Uptake, translocation, size characterization and localization of cerium oxide nanoparticles in radish (*Raphanus sativus* L.). Sci. Total Environ..

[49] Wojcieszek J., Jiménez-Lamana J., Ruzik L., Asztemborska M., Jarosz M., Szpunar J. (2020). Characterization of TiO_2_ NPs in Radish (*Raphanus sativus* L.) by Single-Particle ICP-QQQ-MS. Front. Environ. Sci..

[50] Nair R., Varghese S.H., Nair B.G., Maekawa T., Yoshida Y., Kumar D.S. (2010). Nanoparticulate material delivery to plants. Plant Sci..

[51] Scarpella E., Meijer A.H. (2004). Pattern formation in the vascular system of monocot and dicot plant species. New Phytol..

[52] Schwabe F., Tanner S., Schulin R., Rotzetter A., Stark W., Von Quadt A., Nowack B. (2015). Dissolved cerium contributes to uptake of Ce in the presence of differently sized CeO_2_-nanoparticles by three crop plants. Metallomics.

[53] Ramos S.J., Dinali G.S., Oliveira C., Martins G.C., Moreira C.G., Siqueira J.O., Guilherme L.R.G. (2016). Rare earth elements in the soil environment. Curr. Pollut. Rep..

[54] Liu D., Wang X., Lin Y., Chen Z., Xu H., Wang L. (2012). The effects of cerium on the growth and some antioxidant metabolisms in rice seedlings. Environ. Sci. Pollut. Res..

[55] Rico M., Morales M.I., Barrios A.C., McCreary R., Hong J., Lee W.-Y., Nunez J., Peralta-Videa J.R., Gardea-Torresdey J.L. (2013). Effect of cerium oxide nanoparticles on the quality of rice (*Oryza sativa* L.) grains. J. Agric. Food Chem..

[56] Pošćić F., Mattiello A., Fellet G., Miceli F., Marchiol L. (2016). Effects of cerium and titanium oxide nanoparticles in soil on the nutrient composition of barley (*Hordeum vulgare* L.) kernels. Int. J. Environ. Res. Pub. Health.

[57] Rico M., Johnson M.G., Matthew M.A., Andersen C.P. (2017). Intergenerational responses of wheat (*Triticum aestivum* L.) to cerium oxide nanoparticles exposure. Environ. Sci. Nano.

[58] Zhao L., Peralta-Videa J.R., Rico C.M., Hernandez-Viezcas J.A., Sun Y., Niu G., Servin A., Nunez J.E., Duarte-Gardea M., Gardea-Torresdey J.L. (2014). CeO_2_ and ZnO nanoparticles change the nutritional qualities of cucumber (*Cucumis sativus*). J. Agric. Food. Chem..

[59] Gui X., He X., Ma Y., Zhang P., Li Y., Ding Y., Yang K., Li H., Rui Y., Chai Z. (2015). Quantifying the distribution of ceria nanoparticles in cucumber roots: The influence of labeling. RSC Adv..

[60] Peralta-Videa R., Hernandez-Viezcas J.A., Zhao L., Diaz B.C., Ge Y., Priester J.H., Holden P.A., Gardea-Torresdey J.L. (2014). Cerium dioxide and zinc oxide, nanoparticles alter the nutritional value of soil cultivated soybean plants. Plant Physiol. Biochem..

[61] Elhawat N., Alshaal T., Hamad E., El-Nahrawy E., El-Dein Omara A., El-Nahrawy S., Elsakhawy T., Ghazi A., Abdalla N., Domokos-Szabolcsy E., Faisal M., Saquib Q., Alatar A., Al-Khedhairy A. (2018). Nanoparticle-associated phytotoxicity and abiotic stress under agroecosystems. Phytotoxicity of Nanoparticles.

[62] Jiménez-Lamana J., Wojcieszek J., Jakubiak M., Asztemborska M., Szpunar J. (2016). Single particle ICP-MS characterization of platinum nanoparticles uptake and bioaccumulation by *Lepidium sativum* and *Sinapis alba* plants. J. Anal. At. Spectrom..

[63] US EPA (1995). EPA Method 3052. Microwave assisted acid digestion of siliceous and organically based matrices in US Environmental Protection Agency. Test Methods for Evaluating Solid Waste.

