# Supplementary material for: Influence of Cerium Oxide Nanoparticles on Two Terrestrial Wild Plant Species
Source: Plants (Basel). 2021 Feb 10;10(2):335. doi: 10.3390/plants10020335 (PMC7916331; doi:10.3390/plants10020335)
Supplement: Supplementary file 1 [file plants-10-00335-s001.pdf]

plants

MDPI

Supplementary materials

Influence of cerium oxide nanoparticles on two terrestrial wild plant species

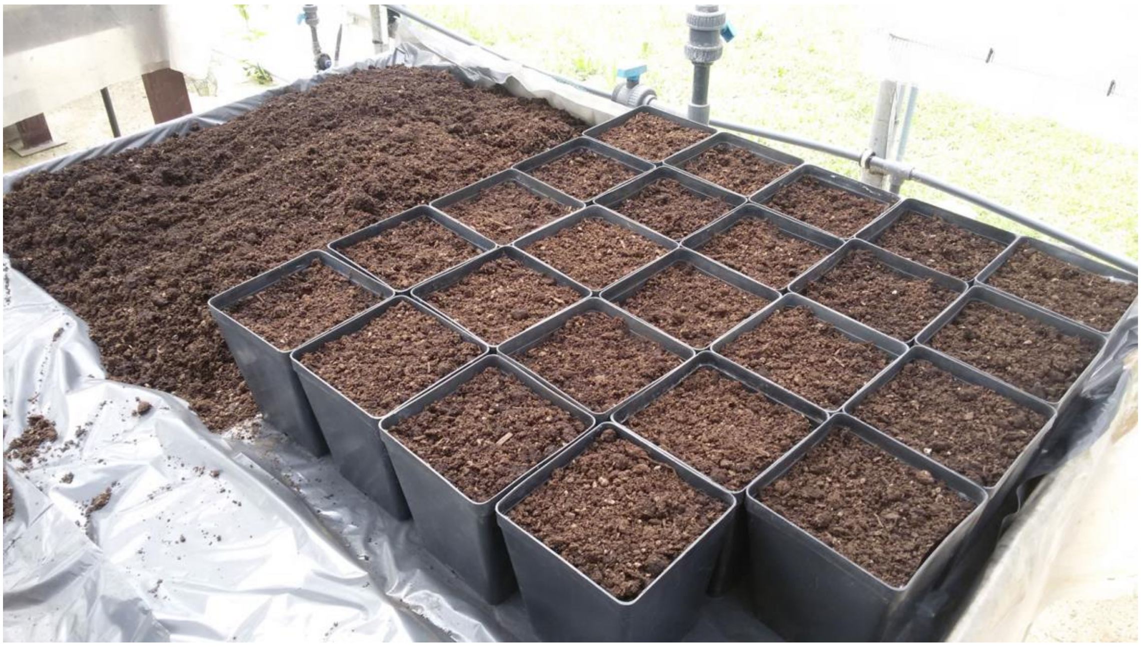

Figure S1 – Preparation of the experiment: filling the pots with the *n*CeO_2 amended substrate.

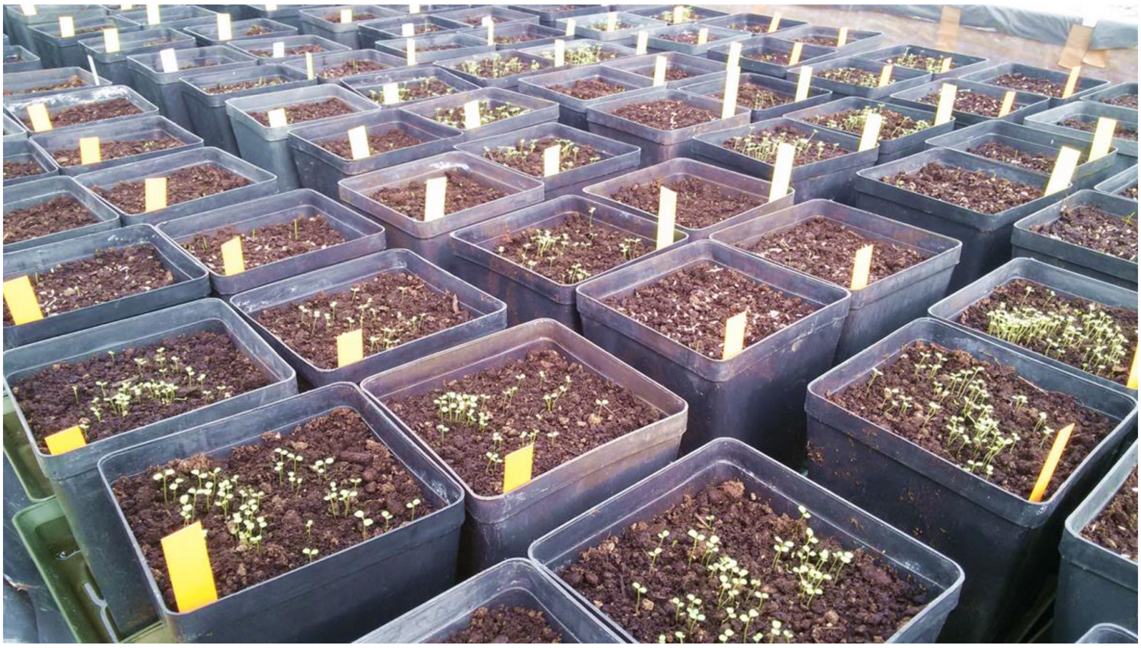

Figure S2 – Plantlets of *H. lanatus* and *D. tenuifolia* 10 d after sowing.

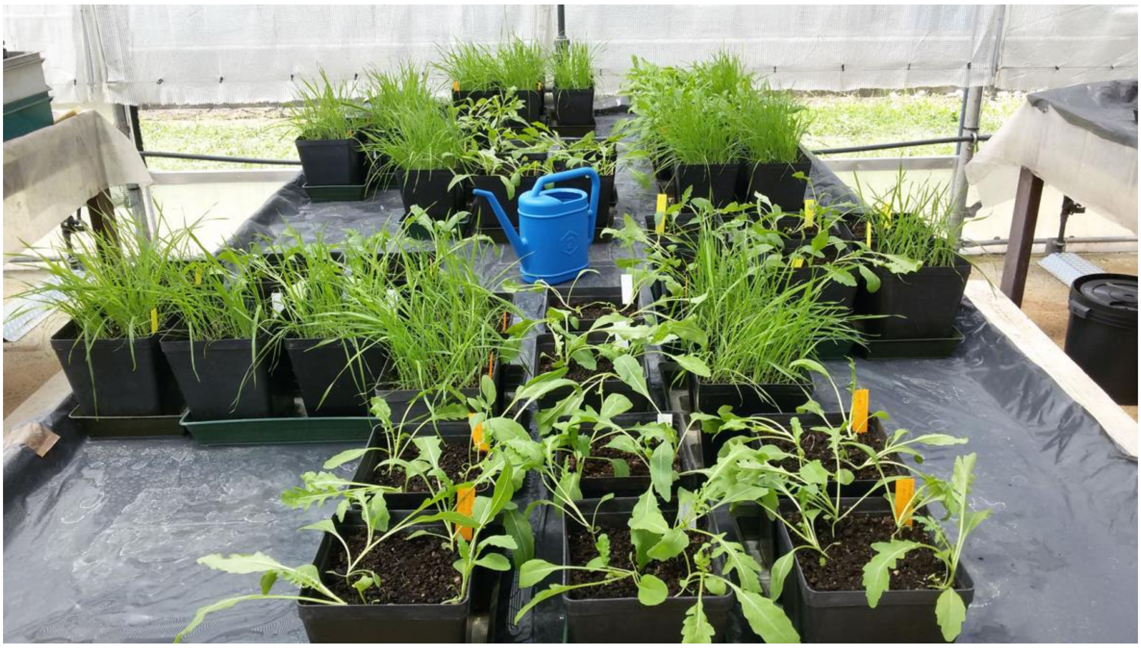

Figure S3 – Plants of *H. lanatus* and *D. tenuifolia* 30 day after sowing.

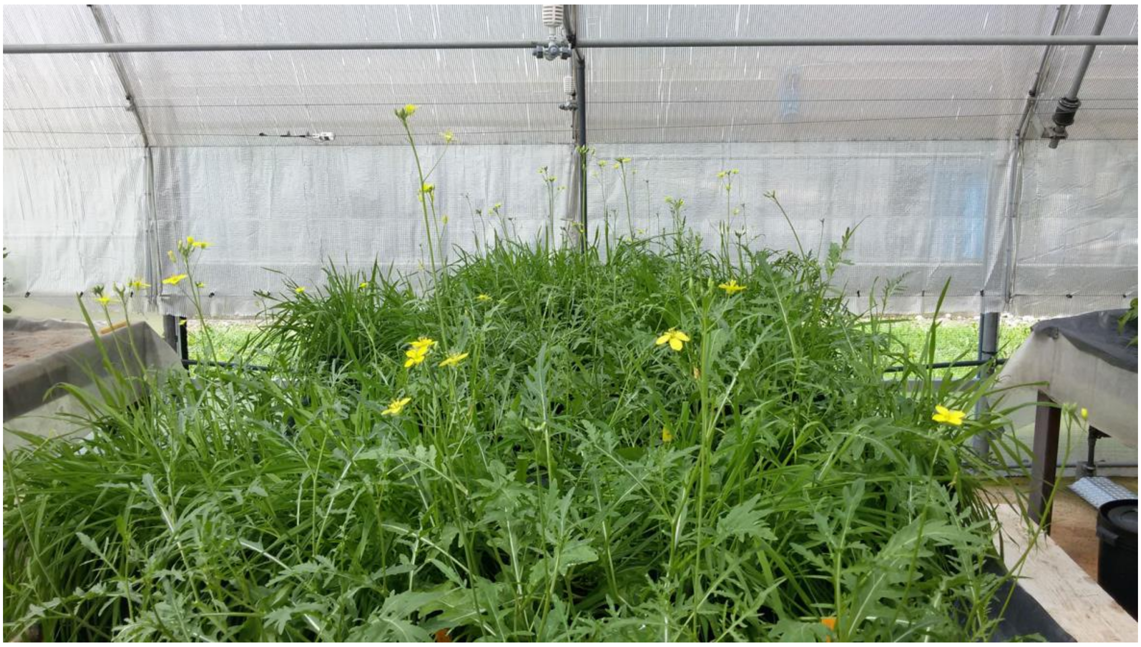

Figure S4 – Plants of *H. lanatus* (in the background) and *D. tenuifolia* before biomass harvesting.

1. Detection of *n*CeO_2 in plant fractions

Small portions (0.03 g) of fresh roots and leaves were harvested, rinsed three times with deionized water and homogenized with 8 mL of 2 mM citrate buffer at pH 4.5, using an ultrasonic bath for 5 minutes. After the homogenization, for every sample 2 mL of the enzyme solution (0.05 g of enzyme dissolved in 2 ml of MilliQ water) were added. The final supernatants were analyzed via single particle inductively coupled plasma mass spectrometry (sp-ICP-MS) NexION 350 (Perkin Elmer, Waltham, MA, USA) to obtain the size distribution of nCeO2.

Table S1 – Most frequent particle size, mean particle size, number of peaks and content of dissolved Ce determined by sp–ICP–MS analysis after enzymatic extraction on roots and leaves of *H. lanatus* and *D. tenuifolia* treated with *n*CeO_2 200 mg L^{-1}.

Species	Plant fraction	*n*CeO_2 size (nm)	Most frequent *n*CeO_2 size (nm)	Mean *n*CeO_2 size (nm)	Pulses (n)	Dissolved Ce (μg L^{-1})
H. lanatus	Roots	25	30 ± 1.45	36 ± 1.34	5785 ± 257	0.27 ± 0.03
	Roots	50	51 ± 1.53	56 ± 1.65	1327 ± 49	7.07 ± 1.10
	Leaves	25	23 ± 1.20	28 ± 1.84	1124 ± 64	0.14 ± 0.01
	Leaves	50	30 ± 0.58	36 ± 1.14	1140 ± 73	0.24 ± 0.05
D. tenuifolia	Roots	25	50 ± 3.46	53 ± 3.35	11,909 ± 711	14.57 ± 1.13
	Roots	50	79 ± 0.88	82 ± 0.87	2855 ± 76	100.30 ± 1.45
	Leaves	25	19 ± 1.20	26 ± 0.51	818 ± 29	0.05 ± 0.02
	Leaves	50	25 ± 0.33	32 ± 0.84	1208 ± 24	0.13 ± 0.01

2. Plant biomass allocation patterns

Experimental biometric dataset was used to evaluate biomass allocation patterns to roots, stems and leaves of studies species in response to nCeO2 treatments.

Table S2 – Two-way ANOVA p value determined for biometric variables of *H. lanatus* and *D. tenuifolia*. ns is not significant at p≤.05, *, ** and *** indicate significance at p≤.05, p≤.01 and p≤.001, respectively.

Source	Roots DW	n. Stems	Stems DW	Leaf area	Leaves DW	Total DW
Species	.0000 ***	.0000 ***	.0000 ***	.0000 ***	.9552 ns	.0123 *
Treatment	.3394 ns	.0094 **	.0574 ns	.0005 ***	.0482 *	.2017 ns
Species x Treatment	.0045 **	.0157 *	.0670 ns	.0958 ns	.6577 ns	.1859 ns

Table S3 – Biomass allocation variables calculated from plant measurements (Poorter et al, 2011).

Variable	Abbreviation	Definition	Unit
Root Mass Fraction	RMF	Root dry mass/Total plant dry mass	g g^{-1}
Stem Mass Fraction	SMF	Stem dry mass/Total plant dry mass	g g^{-1}
Leaf Mass Fraction	LMF	Leaf dry mass/Total plant dry mass	g g^{-1}
Shoot to Root ratio	S/R ratio	(Leaf + Stem dry mass)/Root dry mass	g g^{-1}
Leaf Area Ratio	LAR	Leaf area/Total plant dry mass	m^2 kg^{-1}
Specific Leaf Area	SLA	Leaf area/Leaf dry mass	m^2 kg^{-1}

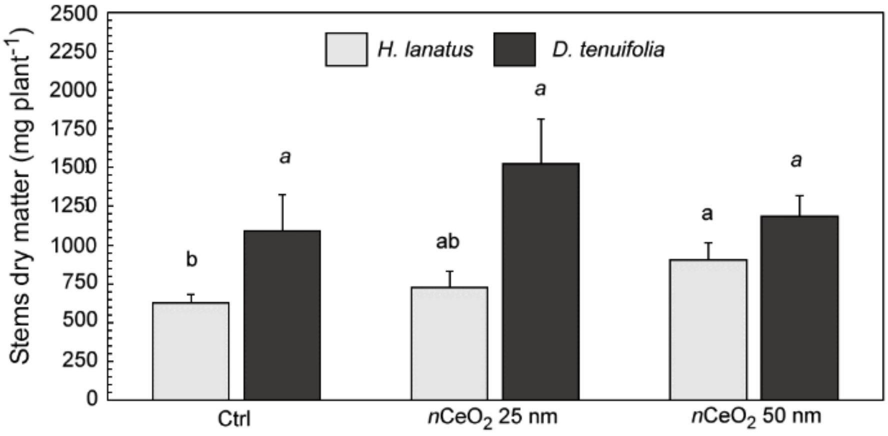

Figure S5. Stems dry matter ± standard deviation of *H. lanatus* and *D. tenuifolia*. Comparison between control and plants grown in presence of 200 mg kg-1 nCeO2 having respectively 25 nm and 50 nm. For each species the statistically significant difference ($p \leq 0.05$) between treatments is indicated by the letters using one-way ANOVA followed by Tukey's test.

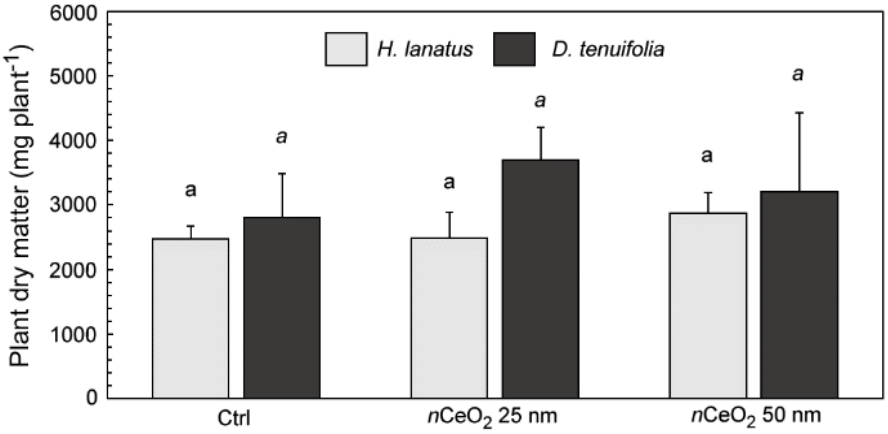

Figure S6. Total plant dry matter ± standard deviation of *H. lanatus* and *D. tenuifolia*. Comparison between control and plants grown in presence of 200 mg kg-1 nCeO2 having respectively 25 nm and 50 nm. For each species the statistically significant difference ($p \leq 0.05$) between treatments is indicated by the letters using one-way ANOVA followed by Tukey's test.

Table S4 – Two-way ANOVA p value determined for biometric ratios calculated for *H. lanatus* and *D. tenuifolia*. ns is not significant at p≤.05, *, ** and *** indicate significance at p≤.05, p≤.01 and p≤.001, respectively.

Source	Root:Shoot	RMF	SMF	LMF	LAR	SLA
Species	.0000 ***	.0000 ***	.0000 ***	.0000 ***	.0000 ***	.0000 ***
Treatment	.0038 **	.0070 **	.1022 ns	.0618 ns	.0021 **	.0017 **
Species x Treatment	.0026 **	.0035 **	.0174 *	.0549 ns	.1134 ns	.0583 ns

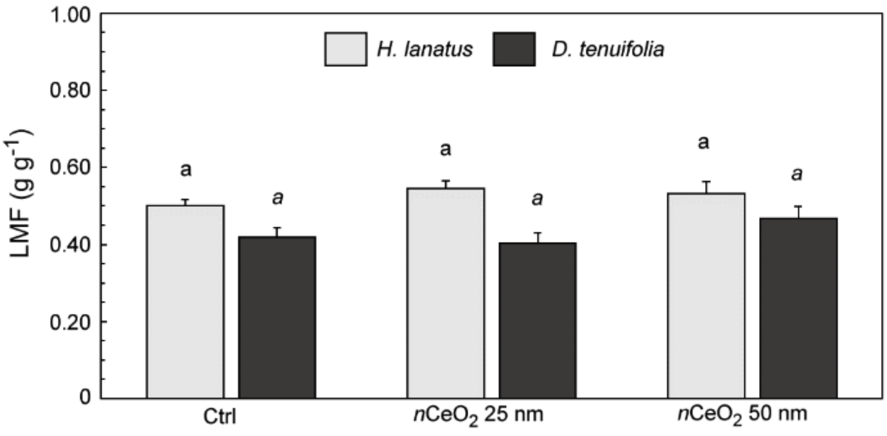

Figure S7. Leaf mass fraction ± standard deviation of *H. lanatus* and *D. tenuifolia*. Comparison between control and plants grown in presence of 200 mg kg-1 nCeO2 having respectively 25 nm and 50 nm. For each species the statistically significant difference ($p \leq 0.05$) between treatments is indicated by the letters using one-way ANOVA followed by Tukey's test.

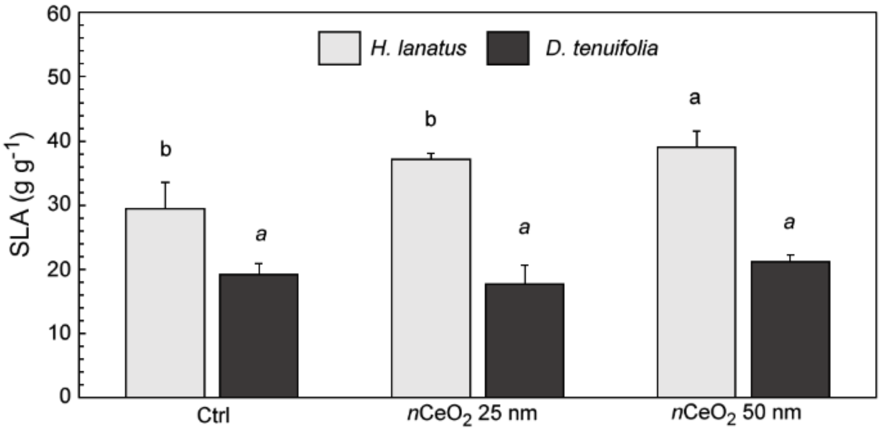

Figure S8 – Specific leaf area* ± standard deviation of *H. lanatus* and *D. tenuifolia*. Comparison between control and plants grown in presence of 200 mg kg-1 nCeO2 having respectively 25 nm and 50 nm. For each species the statistically significant difference ($p \leq 0.05$) between treatments is indicated by the letters using one-way ANOVA followed by Tukey's test. * According to Evans (1972) SLA is the total leaf area of a plant divided by the total leaf weight. This ratio has a relevant ecological importance as describes the allocation of leaf biomass relative to leaf area which in turns refers to carbon gain relative to water loss, within a plant canopy (Gunn et al., 1999).

3. Cerium concentration in plant fractions

Table S5 – Two-way ANOVA p value determined for Ce concentration in plant fractions of *H. lanatus* and *D. tenuifolia*. ns is not significant at p≤.05, *, ** and *** indicate significance at p≤.05, p≤.01 and p≤.001, respectively.

Source	Ce root	Ce stems	Ce leaves
Species	.0289 *	0.2395 ns	.9910 ns
Treatment	.0000 ***	0.0131 *	.0003 ***
Species x Treatment	.1651 ns	.0998 ns	.0020 **

4. Macronutrient and micronutrient concentration in plant fractions

Table S6 – Two-way ANOVA p value for concentration of macronutrients and micronutrients in roots of *H. lanatus* and *D. tenuifolia*. ns is not significant at p≤.05, *, ** and *** indicate significance at p≤.05, p≤.01 and p≤.001, respectively.

Source	K	Mg	Na	P	Cu	Fe	Mn	Zn
Species	.0000 ***	.0000 ***	.0076 **	.0000 **	.0000 ***	.0000 ***	.0000 ***	.0000 ***
Treatment	.4124 ns	.3942 ns	.0044 **	.2220 ns	.8510 ns	.0013 **	.0058 **	.0650 ns
Species x Treatment	.1045 ns	.0671 ns	.5601 ns	.1701 ns	.8797 ns	.1353 ns	.0917 ns	.0000 ***

Table S7 – Two-way ANOVA p value for concentration of macronutrients and micronutrients in stems of *H. lanatus* and *D. tenuifolia*. ns is not significant at p≤.05, *, ** and *** indicate significance at p≤.05, p≤.01 and p≤.001, respectively.

Source	K	Mg	Na	P	Cu	Fe	Mn	Zn
Species	.0004 ***	.1435 ns	.0009 ***	.0198	.0008 ***	.0289 *	.0000 ***	.0108 *
Treatment	.2437 ns	.9615 ns	.1697 ns	.2452 ns	.8216 ns	.0075 **	.0495 *	.4795 ns
Species x Treatment	.4800 ns	.6225 ns	.2653 ns	.7548 ns	.3758 ns	.4410 ns	.0612 ns	.8050 ns

Table S8 – Two-way ANOVA p value for concentration of macronutrients and micronutrients in leaves of *H. lanatus* and *D. tenuifolia*. ns is not significant at p≤.05, *, ** and *** indicate significance at p≤.05, p≤.01 and p≤.001, respectively.

Source	K	Mg	Na	P	Cu	Fe	Mn	Zn
Species	.0115 *	.0000 ***	.2653 ns	.3579 ns	.1970 ns	.6790 ns	.0000 ***	.0000 ***
Treatment	.1777 ns	.8807 ns	.0876 ns	.2470 ns	.0132 *	.1282 ns	.1798 ns	.2486 ns
Species x Treatment	.0442 *	.3137 ns	.2396 ns	.0864 ns	.0947 ns	.0466 *	.1510 ns	.3278 ns

References

Evans, G.C. The quantitative analysis of plant growth. Oxford, UK: Blackwell Scientific Publications. 1972. https://doi.org/10.2307/2259048.

Gunn, S.; Farrar, J.F.; Collis, B.E.; Nason, M. Specific leaf area in barley: individual leaves versus whole plants. *New Phytol.*, 1999, 143, 45–51. https://doi.org/10.1046/j.1469-8137.1999.00434.x.

Poorter, H.; Niklas, K.J.; Reich, P.B.; Oleksyn, J.; Poot, P.; Mommer, L.; Biomass allocation to leaves, stems and roots: meta-analyses of interspecific variation and environmental control. *New Phytol.*, 2012, 193: 30–50. http://doi.org/10.1111/j.1469-8137.2011.03952.x.
